# A Systemic Review and Meta-analysis of the Effect of SARS-CoV-2 Infection on Sperm Parameters

**DOI:** 10.34133/2022/9835731

**Published:** 2022-07-13

**Authors:** Xi Chen, Jinli Ding, Miao Liu, Kai Xing, Peng Ye, Junxia Min, Yan Zhang, Tailang Yin

**Affiliations:** ^1^ Reproductive Medical Centre, Renmin Hospital of Wuhan University, Wuhan, China; ^2^ Department of Pediatrics, Renmin Hospital of Wuhan University, Wuhan, China; ^3^ Department of Cardiovascular Surgery, Renmin Hospital of Wuhan University, Wuhan, China; ^4^ Department of Pharmacy, Renmin Hospital of Wuhan University, Wuhan, China; ^5^ The First Affiliated Hospital, Institute of Translational Medicine, Zhejiang University School of Medicine, Hangzhou, China; ^6^ Department of Clinical Laboratory, Renmin Hospital of Wuhan University, Wuhan, China

## Abstract

*Objective*. Several studies examined the putative effects of SARS-CoV-2 infection on sperm parameters. However, the results remain controversial. In this study, we conducted the most up-to-date systematic review and meta-analysis to investigate the effect of SARS-CoV-2 infection on sperm quality in COVID-19-positive and COVID-19-negative male participants. *Method*. Seven databases were searched for literature released through June 10, 2022, containing estimates for the outcomes of interest. Using a random-effects model (REM) or a fixed-effects model (FEM), we analyzed the pooled results. The quality of all included studies was assessed by the Newcastle-Ottawa scale. In addition, we performed a quantitative and subgroup analysis of semen data across all included studies. *Results*. Fourteen studies were extracted from 10 publications, involving a total of 1174 participates for meta-analysis. Sperm parameters of 521 COVID-19 male patients and 653 controls were analyzed. In 8 case-control studies, the pooled mean difference (MD) of total sperm motility was -5.37% (95% confidence interval (CI): -8.47 to -2.28; p<0.05 ), suggesting that total motility was significantly impaired in male COVID-19 cases. Subgroup analysis showed a significant decrease in semen volume, sperm concentration, and total motility in 238 patients with a recovery time of less than 90 days. Moreover, in the other 6 included pre- to post-COVID-19 studies, the pooled MDs of sperm concentration, total sperm count, total motility, progressive motility, and normal morphology were −6.54×10 ^6^/ml (95% CI: -10.27 to -2.81; p<0.05 ), −38.89×10 ^6^ (95% CI: -59.20 to -18.58; p<0.05 ), -7.21% (95% CI: -14.36 to -0.07; p<0.05 ), -5.12% (95% CI: -8.71 to -1.53; p<0.05 ), and -1.52% (95% CI: -2.88 to -0.16; p<0.05 ), respectively, which indicate SARS-CoV-2 infection significantly affected these five sperm parameters. *Conclusion*. Our results revealed that SARS-CoV-2 infection was significantly correlated with decreased sperm quality. Of six sperm parameters, total motility and sperm concentration were the most significantly decreased parameters. These results suggest a possible negative influence of SARS-CoV-2 infection on testicular function and male fertility. Given the potential detrimental effect of SARS-CoV-2 on semen quality, male reproductive health should be monitored closely in patients with COVID-19. This trial is registered with CRD42021275823.

## 1. Introduction

In December 2019, the coronavirus disease 2019 (COVID-19) was initially identified in Wuhan, China, leading to a worldwide pandemic and mass panic [ 1]. Now, the COVID-19 epidemic remains critical, with new cases being reported every day around the world. The disease was caused by the severe acute respiratory syndrome coronavirus 2 (SARS-CoV-2), a type of enveloped, positive-sense single-stranded RNA. SARS-CoV-2 shares many common features with SARS-CoV-1, which caused a large epidemic in 2002-2003, and is similarly transmitted primarily by respiratory droplets and close contact [ 2, 3].

SARS-CoV-2 infection primarily affects the respiratory system, with patients presenting with fever, cough, nasal congestion, sore throat, myalgia, etc. Some patients may experience loss of taste or smell, or shortness of breath in severe cases [ 4, 5]. In addition, it is found that some preexisting chronic diseases such as hypertension and diabetes are strongly associated with increased disease severity in patients with COVID-19 [ 6]. One study reported that orchitis was detected in the testes of male patients who died of COVID-19 by biopsy [ 7], revealing that the testis might be affected by COVID-19. SARS-CoV-2 virus is known to invade cells primarily based on the expression of a functional receptor angiotensin-converting enzyme 2 (ACE2) and a cellular protease, transmembrane protease serine 2 (TMPRSS2) [ 8– 10]. Previous studies have demonstrated that spermatozoa, Leydig cells, and Sertoli cells in the seminiferous ducts have high expression levels of ACE2 proteins, implying that the testis is a potential infectious organ for SARS-CoV-2 [ 11– 14].

There are studies showed that SARS-CoV-2 infection can impair male fertility and the genital system [ 15– 20]. However, the specific effects of SARS-CoV-2 infection on sperm parameters have not been demonstrated until now. Accurate assessment of the impact of SARS-CoV-2 infection on semen quality is very important for the fertility prognosis of male patients with COVID-19, and it can arouse the attention of COVID-19 patients and reproductive clinicians and further strengthen regular monitoring and timely intervention measures for COVID-19 male patients. In this meta-analysis, we compared the semen quality between COVID-19 (+) male patients and COVID-19 (-) male participants. The primary purpose of this systematic and meta-analysis was to compare the sperm parameters in the two groups including semen volume, total sperm count, sperm concentration, total sperm motility, progressive sperm motility, and sperm morphology. We also summarized the presence or absence of SARS-CoV-2 RNA in semen and the pathological findings of the testes in COVID-19 male patients and further analyzed its potential adverse effect on male fertility.

## 2. Results

### 2.1. Study Characteristics

**Figure 1:**
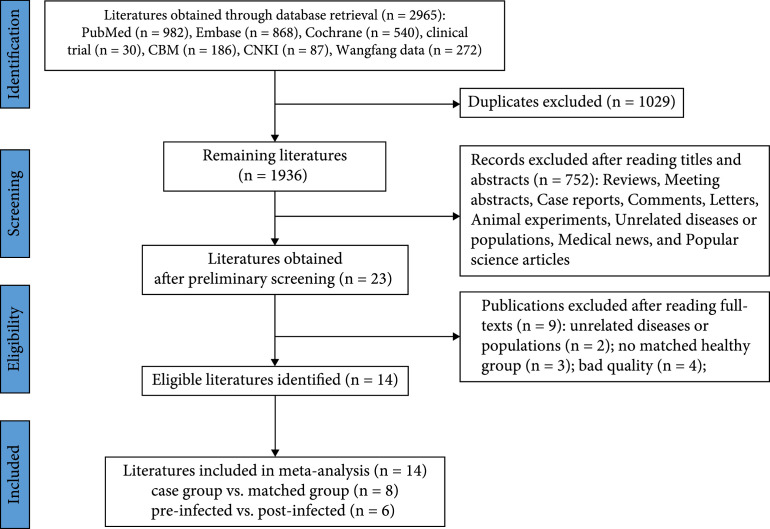
The PRISMA flow diagram of the literature screening.

Of 2965 articles identified from the database search, 23 articles were selected for full-text reading. After excluding 2 studies for not providing applicable data on semen quality, 3 articles for no matched control group, and 4 articles for bad quality, we eventually included 14 studies for meta-analysis [ 21– 34], including 8 articles that compared the semen quality between the COVID-19 case group and age-matched control group, and 6 articles comparing patients’ sperm parameters before and after COVID-19 infection. The PRISMA (Preferred Reporting Items for Systematic Review and Meta-analysis Protocols) flow diagram of the literature screening is presented in Figure 1. The quality assessment showed that all 14 studies enrolled in the quantitative analysis were of good quality (Supplementary Table S2).

The characteristics of the 14 studies included in the meta-analysis are presented in Table 1. Almost all COVID-19 patients in the included studies were reported to have recovered from the disease during the semen analysis. The semen analysis in 11 studies was performed following WHO 2010 criteria [ 21– 24, 26– 29], and another 2 studies also adopted the WHO standard, but its version was not informed [ 25, 30]. One study did not explain the guideline on which semen analysis was based, but it is stated that all tests were performed in an EQUAS-certified laboratory [ 34].

**Table 1:** Characteristics of the 14 studies included in the meta-analysis.

Study	Country	Study design	Cohort ( n )	Control ( n )	Age of cohort (years)	Quality (NOS)	Main conclusion
Temiz 2020 [ 21]	Turkey	Prospective cross-sectional studies	10	10	37.00±8.69	9	NSM
Ruan 2020 [ 22]	China	Descriptive case series	55 (55R; 7Mi, 24Mo, 24S)	145	34.29±2.54(Mi), 31.25 ± 1.09(Mo), 29.79±0.93(S)	9	SC, TSC, TM
Maleki 2021 [ 26]	Germany	Prospective longitudinal cohort study	84 (84R; 1Mi, 23Mo, 27S, 33C)	105	34.7±6.3	8	SV, PM, NSM, SC, TSC
Guo 2021 [ 27]	China	Prospective cohort study	41 (41R)	50	26.0 (IQR: 22.0~34.0)	9	TSC, SC, PM, TM
Best 2021 [ 29]	USA	Prospective cohort study	30 (30R)	30	40 (IQR: 24.75)	9	SC, TSC
Holtmann 2020 [ 25]	Germany	Pilot cohort study	18 (18R; 14Mi, 4Mo)	14	42.7±10.4(Mi), 40.8±8.7(Mo)	7	SC, TSC, PM, TM
Hu 2022 [ 32]	China	Prospective cohort study	36 (36R; 4Mi, 17Mo, 15S)	45	31.75±5.77	8	—
Enikeev 2022 [ 34]	Russia	Prospective cohort study	37 (37R)	44	46.7±9.9	7	—
Pazir 2021 [ 23]	Turkey	Prospective cohort study	24 (24R; 24Mi)	24	34.7±6.4	9	TM
Koç 2021 [ 24]	Turkey	Prospective cohort study	21 (21R)	21	32±6.30	7	SV, PM, NSM
Erbay 2021 [ 28]	Turkey	Prospective observational study	69 (69R; 26Mi, 43Mo)	69	30.4±4.8(Mi), 31.06±4.2(Mo)	7	TM
Hamarat 2022 [ 31]	Turkey	Prospective cohort study	41 (41R; 39Mi, 2Mo)	41	31.29±5.95	7	SC, TSC
Wang 2022 [ 33]	China	Retrospective cohort study	26 (26R)	26	33.67±4.58	7	NSM
Gul 2021 [ 30]	Turkey	Cross-sectional analysis	29 (29R)	29	31.21±5.48	9	—

R: recovery phase; Mi: mild; Mo: moderate; S: severe; C: critical. It indicates the disease stage and severity of the COVID-19 patients, and we do not give the full extent of this information because some of it is not available in the original article. “Main Conclusion” mainly presents the sperm parameters decreased in the COVID-19 patient group compared with the control group—SV: semen volume; SC: sperm concentration; TSC: total sperm count; TM: total motility; PM: progressive motility; NSM: normal sperm morphology; “-” indicates no significant differences, which suggests all sperm parameters were comparable between the COVID-19 group and the control group. All data are presented as mean±SD unless indicated.

In Table 2, we sorted out and listed original data condition of the 14 included studies. We found that these articles reached different results. As shown in Table 1, Temiz et al. observed that COVID-19 infection significantly reduced sperm morphology [ 21]. Best et al. found that total sperm count and sperm concentration were significantly lower in COVID-19 cases compared to healthy men [ 29]. It is reported that multiple sperm parameters, such as progressive motility, total sperm number, and semen volume, were decreased in COVID-19 male patients compared to the uninfected controls [ 22– 28]. Interestingly, a subset of COVID-19 patients recovered for more than 3 months, and their sperm quality was comparable to that of COVID-19 negative male control participants [ 30, 32– 34].

**Table 2:** Outcomes reported in the 14 studies included in the meta-analysis.

Study	Semen volume	Sperm concentration	Total sperm count	Total motility	Progressive motility	Morphology
Temiz 2020 [ 21]	m	a	a	NA	m	m
Ruan 2020 [ 22]	a	a	a	a	a	NA
Maleki 2021 [ 26]	a	a	a	NA	a	a
T.Guo 2021 [ 27]	m	m	m	m	m	m
Best 2021 [ 29]	m	m	m	NA	NA	NA
Holtmann 2020 [ 25]	a	a	a	a	a	NA
Hu 2022 [ 32]	a	m	m	m	m	NA
Enikeev 2022 [ 34]	a	a	a	a	a	a
Pazir 2021 [ 23]	a	a	NA	a	a	NA
Koç 2021 [ 24]	m	m	m	a	a	m
Erbay 2021 [ 28]	a	a	a	a	a	NA
Hamarat 2022 [ 31]	a	m	m	a	a	a
Wang 2022 [ 33]	m	m	m	m	m	m
Gul 2021 [ 30]	a	a	a	a	a	NA

a : average/mean and SD; m : median and IQR/range; NA: not reported. Data of Maleki 2021 [ 26] was extracted from histograms in a figure.

### 2.2. Total Motility Was Significantly Reduced in Male COVID-19 Cases

In the 8 included case-control studies, semen data on 311 COVID-19 male patients and 443 healthy controls were analyzed [ 21, 22, 25– 27, 29, 32, 34]. All these studies provided information on semen volume, total sperm count, and sperm concentration. Seven articles provided data on progressive motility, whereas six articles reported data on total motility, and four studies provided data on sperm morphology. We conducted a meta-analysis to examine whether semen quality was significantly affected in COVID-19 cases. Results of the meta-analysis and subgroup analysis are shown in Table 3.

**Table 3:** Summary of the meta-analysis of the 14 studies included in this study.

Sperm parameters	No. of studies	MD (95% CI)	Heterogeneity			Publication bias	
			$I^{2}$	H test	P value of Q statistics	Egger’s test	Begg’s test
*Case to control*							
Semen volume	8	-0.49 (-1.03, 0.05)	87.19	7.81	<0.001	0.1518	1.0985
<90d	6	-19.82 (-38.49, -1.16)	92.20	12.83			
>90d	2	12.39 (-16.23, 41.01)	62.65	2.68			
Sperm concentration	8	-11.24 (-29.93, 7.45)	93.93	16.47	<0.001	0.0760	1.4638
<90d	6	-19.82 (-38.49, -1.16)	92.20	12.83			
>90d	2	12.39 (-16.23, 41.01)	62.65	2.68			
Total sperm count	8	-61.33 (-128.46, 5.80)	95.23	20.97	<0.001	0.1428	1.4638
<90d	6	-71.67 (-149.98, 6.64)	96.03	25.16			
>90d	2	-40.98 (-87.16, 5.21)	0.00	1.00			
Total motility	6	-5.37 (-8.47, -2.28)	23.53	1.31	0.26	0.1499	0.2597
<90d	4	-6.71 (-10.67, -2.75)	16.69	1.20			
>90d	2	-3.42 (-7.75, 0.90)	7.54	1.08			
Progressive motility	7	-5.98 (-16.03, 4.07)	94.80	19.22	<0.001	0.6295	1.4520
<90d	5	-7.47 (-22.57, 7.64)	96.39	27.74			
>90d	2	-2.39 (-6.40, 1.61)	0.00	1.00			
Morphology	4	-4.05 (-9.72, 1.61)	97.61	41.81	<0.001	0.9920	1.2659
<90d	3	-3.93 (-10.51, 2.65)	98.41	62.72			
*Pre to post*							
Semen volume	6	-0.18 (-0.40, 0.04)	26.42	1.36	0.65	0.4562	0.7071
<90d	2	-0.32 (-0.75, 0.10)	0.00	0.23			
>90d	4	-0.13 (-0.39, 0.13)	49.75	1.99			
Sperm concentration	6	-6.54 (-10.27, -2.81)	4.67	1.05	0.39	0.9355	1.2929
<90d	2	-12.53 (-19.82, -5.23)	0.00	0.44			
>90d	4	-4.43 (-8.77, -0.08)	0.00	0.44			
Total sperm count	5	-25.35 (-55.65, 4.95)	56.03	2.27	0.34	0.8396	1.1935
<90d	2	-54.86 (-79.37, -30.35)	0.00	1.00			
>90d	3	-8.64 (-37.65, 20.36)	21.19	1.27			
	4 ^s^	-38.89 (-59.20, -18.58)	10.90	1.12		0.9122	1.2659
Total motility	6	-7.21 (-14.36, -0.07)	82.43	5.69	<0.001	0.6863	1.0000
<90d	2	-7.13 (-17.10, 2.85)	50.11	2.00			
>90d	4	-6.85 (-16.57, 2.87)	87.81	8.21			
Progressive motility	6	-5.12 (-8.71, -1.53)	50.41	2.02	0.36	0.7486	0.4524
<90d	2	-6.91 (-16.80, 2.99)	59.22	2.45			
>90d	4	-4.67 (-9.16, -0.17)	60.32	2.52			
Morphology	3	-1.52 (-2.88, -0.16)	68.69	3.19	0.04	0.3089	1.7037
<90d	2	-0.75 (-1.17, -0.33)	0.00	1.00			

Note: the superscript “s” means the revised results after sensitivity analysis indicating one paper should be removed in this meta-analysis of the parameter.

#### 2.2.1. Semen Volume

Eight studies provided data on semen volume [ 21, 22, 25– 27, 29, 32, 34]. The results showed that semen volume was not significantly different between the COVID-19 male patients and the COVID-19 (-) control cohort (REM, MD: -0.49 ml; 95% CI: -1.03 to 0.05; p>0.05 ). Our subgroup analysis based on the recovery time showed that semen volume was significantly decreased in the COVID-19 group whose recovery time was less than 90 days (REM, MD: -19.82 ml; 95% CI: -38.49 to -1.16; p<0.05 ), and no significant difference was observed in the control group and the COVID-19 group whose recovery time was more than 90 days (REM, MD: 12.39 ml, 95% CI: -16.23 to 41.01; p>0.05 ).

#### 2.2.2. Sperm Concentration

Eight studies provided data on sperm concentration [ 21, 22, 25– 27, 29, 32, 34]. The pooled MD in the 8 studies by REM was −11.24×10 ^6^/ml (95% CI: -29.93 to 7.45; p>0.05 ). In the subgroup analysis, the pooled MD in the 6 studies where COVID-19 patients recovered for less than 90 days was −19.82×10 ^6^/ml (REM, 95% CI: -38.49 to -1.16; p<0.05 ). In the other 2 studies with patients who recovered for more than 90 days, the pooled MD was 12.39×10 ^6^/ml (REM, 95% CI: -16.23 to 41.01; p>0.05 ).

#### 2.2.3. Total Sperm Count

Eight studies reported data on total sperm count [ 21, 22, 25– 27, 29, 32, 34]. The pooled MD by REM was −61.33×10 ^6^ (95% CI: -128.46 to 5.80; p>0.05 ). In the subgroup analysis, the pooled MDs of total sperm count in patients recovered for less than 90 days and patients recovered for more than 90 days were −71.67×10 ^6^ (REM, 95% CI: -149.98 to 6.64; p>0.05 ) and −40.98×10 ^6^ (FEM, 95% CI: -87.16 to 5.21; p>0.05 ), respectively. The results showed no significant difference in total sperm count in the case group compared to those control group.

#### 2.2.4. Total Motility

**Figure 2:**
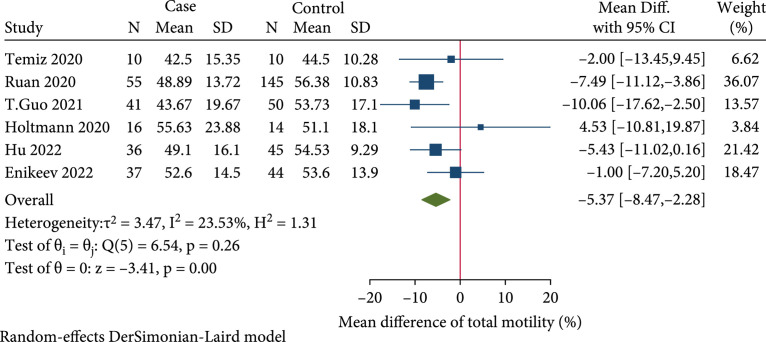
Forest plot showing the effect of SARS-CoV-2 infection on total motility in the case-control studies.

Six studies provided data on total motility [ 21, 22, 25, 27, 32, 34]. Pooled results showed that the COVID-19 cases cohort was shown to have notably lower total motility than the COVID-19 (-) control cohort (FEM, MD: -5.37%; 95% CI: -8.47 to -2.28; p<0.05 ) (Figure 2). Subgroup analysis in the 4 studies with patients’ recovery time less than 90 days also indicated that total motility was significantly reduced in the case group (FEM, MD: -6.71%; 95% CI: -10.67 to -2.75; p<0.05 ). In the other 2 studies with patients’ recovery time more than 90 days, no statistically significant difference in total motility was observed between the two cohorts (FEM, MD: -3.42%; 95% CI: -7.75 to 0.90; p>0.05 ).

#### 2.2.5. Progressive Motility

Seven studies provided data on progressive motility [ 21, 22, 25– 27, 32, 34]. Pooled MD by random-effects model was -5.98% (95% CI: -16.03 to 4.07; p>0.05 ). In the subgroup analysis, the pooled results of progressive motility in patients recovered for less than 90 days and more than 90 days were -7.47% (REM, 95% CI: -22.57 to 7.64; p>0.05 ) and -2.39% (FEM, 95% CI: -6.40 to 1.61; p>0.05 ), respectively. It showed that progressive motility was not significantly different in the COVID-19 case cohort and the COVID-19 (-) control cohort.

#### 2.2.6. Normal Sperm Morphology

Four studies provided data on normal sperm morphology [ 21, 26, 27, 34]. Pooled mean difference by REM was -4.05% (95% CI: -9.72 to 1.61; p>0.05 ). Subgroup analysis showed that there was no significant difference in normal sperm morphology in the COVID-19 cases where patients recovered for less than 90 days (REM, MD: -3.93%; 95% CI: -10.51 to 2.65; p>0.05 ). We did not conduct a meta-analysis of patients recovered for more than 90 days since only one study contains the relevant data.

### 2.3. SARS-CoV-2 Infection Significantly Impaired Sperm Concentration, Total Sperm Count, Total Motility, Progressive Motility, and Normal Sperm Morphology

In the six pre- to post-COVID-19 studies, semen data on 210 participants before and after COVID-19 were included [ 23, 24, 28, 30, 31, 33]. All 6 studies provided data on semen volume, total sperm motility, sperm concentration, and progressive sperm motility, 5 studies reported data on total sperm number, and 3 studies provided information on normal sperm morphology. We performed a quantitative analysis to examine whether sperm quality was significantly decreased after COVID-19 infection. Results of the meta-analysis and subgroup analysis are shown in Table 3.

#### 2.3.1. Semen Volume

Six articles provided data on semen volume [ 23, 24, 28, 30, 31, 33]. Pooled results revealed that semen volume was not significantly influenced by SARS-CoV-2 infection (FEM, MD: -0.18 ml; 95% CI: -0.40 to 0.04; p>0.05 ). In the subgroup analysis, the pooled MDs of semen volume in patients recovered for less than 90 days and more than 90 days were -0.32 ml (FEM, 95% CI: -0.75 to 0.10; p>0.05 ) and -0.13 ml (FEM, 95% CI: -0.39 to 0.13; p>0.05 ), respectively. It also showed semen volume was comparable in patients before and after COVID-19.

#### 2.3.2. Sperm Concentration

**Figure 3:**
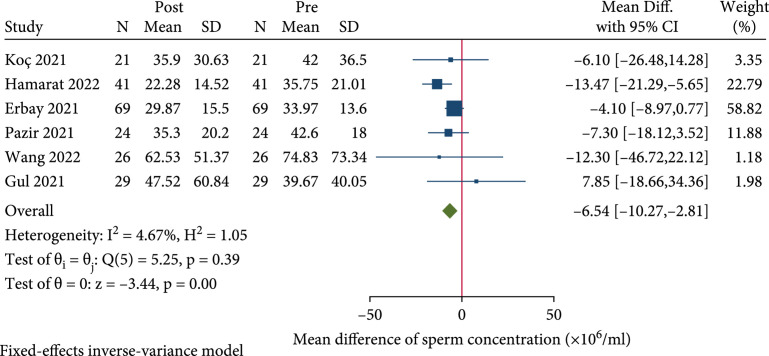
Forest plot showing the effect of SARS-CoV-2 infection on sperm concentration in the pre- to post-COVID-19 studies.

Six articles provided data on sperm concentration [ 23, 24, 28, 30, 31, 33]. Results of quantitative synthesis indicated that sperm concentration was significantly reduced after SARS-CoV-2 infection (FEM, MD: −6.54×10 ^6^/ml; 95% CI: -10.27 to -2.81; p<0.05 ) (Figure 3). In our subgroup analysis, the pooled MDs of sperm concentration in patients recovered for less than 90 days and more than 90 days were −12.53×10 ^6^/ml (FEM, 95% CI: -19.82 to -5.23; p<0.05 ) and −4.43×10 ^6^/ml (FEM, 95% CI: -8.77 to -0.08; p<0.05 ), respectively. It showed a significant reduction in post-COVID-19 groups compared with the pre-infection values regardless of the length of the recovery time.

#### 2.3.3. Total Sperm Count

Five studies provided data on total sperm count [ 24, 28, 30, 31, 33]. Pooled results of these five studies showed that no significant difference in total sperm count was observed before and after SARS-CoV-2 infection (REM, MD: −25.35×10 ^6^; 95% CI: -55.65 to 4.95; p>0.05 ). We conducted sensitivity analysis due to the high heterogeneity ( $I^{2}=56.03\%$ ). The analysis showed that excluding the article by Gul et al. [ 30] remarkably reduced the heterogeneity ( $I^{2}=10.90$ %) (Supplementary Figure S2). Pooled MD of the remaining four studies was −38.89×10 ^6^ (FEM, 95% CI: -59.20 to -18.58; p<0.05 ). In the subgroup analysis, total sperm count showed a significant reduction in the group with patients’ recovery time less than 90 days (FEM, MD: −54.86×10 ^6^; 95% CI: -79.37 to -30.35; p<0.05 ), and no significant change in the group with the recovery time more than 90 days (FEM, MD: −8.64×10 ^6^; 95% CI: -37.65 to 20.36; p>0.05 ).

#### 2.3.4. Total Motility

**Figure 4:**
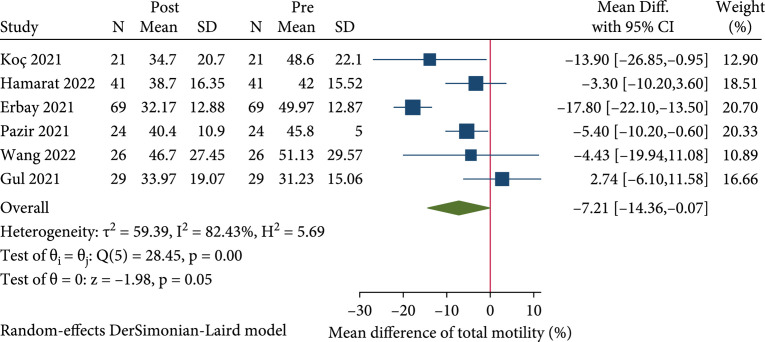
Forest plot showing the effect of SARS-CoV-2 infection on total motility in the pre- to post-COVID-19 studies.

Six studies provided data on total motility [ 23, 24, 28, 30, 31, 33]. Results revealed that total sperm motility was significantly decreased in patients after COVID-19 infection (REM, MD: -7.21%; 95% CI: -14.36 to -0.07; p<0.05 ) (Figure 4). The subgroup analysis showed that no significant difference was observed in total motility in post-infection cohort in both the recovery time>90 days and < 90 day groups. The pooled MDs of total motility in the recovery time>90 days and < 90 days groups were -7.13% (REM, 95% CI: -17.10 to 2.85; p>0.05 ) and -6.85% (REM, 95% CI: -16.57 to -2.87; p>0.05 ), respectively.

#### 2.3.5. Progressive Motility

Six studies provided data on progressive motility [ 23, 24, 28, 30, 31, 33]. Our results indicated that progressive sperm motility was significantly reduced in patients after SARS-CoV-2 infection in comparison to the pre-infection values (REM, MD: -5.12%; 95% CI: -8.71 to -1.53; p<0.05 ). The subgroup analysis showed that no significant difference was observed in progressive motility in patients recovered for less than 90 days (REM, MD: -6.91%; 95% CI: -16.80 to 2.99; p>0.05 ), and a significant decrease was observed in patients recovered for more than 90 days (REM, MD: -4.67%; 95% CI: -9.16 to -0.17; p<0.05 ).

#### 2.3.6. Normal Sperm Morphology

**Figure 5:**
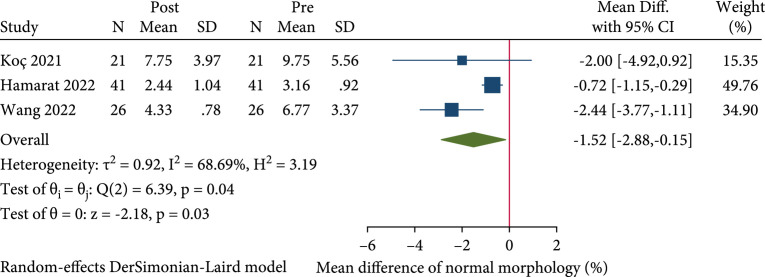
Forest plot showing the effect of SARS-CoV-2 infection on normal morphology in the pre- to post-COVID-19 studies.

Three studies reported data on morphology [ 24, 31, 33]. Pooled results showed that normal morphology was significantly decreased in patients after COVID-19 infection (REM, MD: -1.52%; 95% CI: -2.88 to -0.16; p<0.05 ) (Figure 5). In subgroup analysis, the pooled MD of normal morphology in patients recovered for less than 90 days was -0.75% (REM, 95% CI: -1.17 to -0.33; p<0.05 ). We did not perform quantitative analysis of patients recovered for more than 90 days since only one study contains the relevant data.

No significant publication bias in the funnel plots was observed (Supplementary Figure S1). Sensitivity analyses showed that the results of our meta-analysis were stable and reliable generally (Supplementary Figure S2).

## 3. Discussion

Highly contagious, the COVID-19 outbreak has become a huge threat to human health. Epidemiological research observed that men have a higher susceptibility and mortality to SARS-CoV-2 infection than women [ 35– 37]. The fighting against COVID-19 is ongoing, and we must be conscious of the potential adverse influence of COVID-19 on male health. Some literature reported that COVID-19 infection may have a negative effect on male reproductive health [ 15– 17, 19, 20]. However, this is the most up-to-date systematic review and meta-analysis to evaluate the association between COVID-19 infection and sperm parameters, which included 14 studies with a relatively large number of COVID patients. In addition, we conducted subgroup analysis based on the recovery time.

Among 14 included studies, 8 studies were analyzed by comparing semen quality between COVID-19 recovered patients and their contemporary COVID-19 (-) controls, 6 of which compared sperm quality of the same patients before and after SARS-CoV-2 infection. The number of COVID-19 patients included in each article ranged from 10 to 84, and we finally included 521 COVID-19 patients for quantitative analysis. In the 8 case-control studies, total sperm motility was shown to be significantly lower in COVID-19 recovered groups than those in the controls. Subgroup analysis showed a significant decrease in semen volume, sperm concentration, and total motility in 238 patients with a recovery time of less than 90 days. In the 6 pre- to post-COVID-19 studies, total sperm motility, progressive sperm motility, total sperm count, sperm concentration, and normal morphology were significantly decreased in patients after COVID-19 infection compared to their previous uninfected values.

Based on our meta-analyses, total motility was significantly impaired by SARS-CoV-2 infection in both case-control studies and pre- to post-COVID-19 studies. Subgroup analysis showed that sperm concentration was significantly reduced in patients with a recovery time less than 90 days in both case-control studies and pre- to post-COVID-19 studies. Total sperm motility is the percentage of progressive and nonprogressive motile sperms in all sperms and is closely related to pregnancy rate [ 38]. Sperm concentration refers to the number of sperms per milliliter of semen and is related to the time to pregnancy and the pregnancy rate [ 38]. In conclusion, of the six sperm parameters we studied, five were significantly lower in the post-COVID-19 groups in the 6 pre- to post-COVID-19 studies, and one was significantly lower in the COVID-19 groups in the 8 case-control studies. The decrease of sperm quality in the pre- to post-COVID-19 studies was more pronounced than those of case-control studies.

Furthermore, based on the subgroup analyses, in case to control studies, semen volume, sperm concentration, and total motility were significantly reduced in patients with a recovery time of less than 90 days, while none of the sperm parameters were significantly decreased in patients with a recovery time of more than 90 days. Similarly, in pre- to post-COVID-19 studies, three parameters were decreased significantly in patients with a recovery time less than 90 days, while two parameters were decreased notably in patients with a recovery time of more than 90 days. As the spermatogenesis needs 74 days [ 39], we hypothesize that sperm parameters decrease significantly for approximately three months after COVID-19 infection, and reverted spermatogenesis might be attributed to the improved sperm parameters after 3 months. However, we could not conclude that the effect of COVID-19 infection on sperm parameters was reversible as we did not evaluate the changes in semen quality in the same patients at different recovery times in a large sample size.

In this study, we performed subgroup analyses based on whether the patients’ recovery time was greater than 90 days. It is primarily to observe the long-term effects of COVID-19 infection on male patients. Besides, it has been suggested that it is more objective to see changes in sperm quality 3 months after infection due to the cycle of spermatogenesis [ 22, 25, 30, 40]. Moreover, the patients’ age, severity of disease, and medication are also important factors affecting semen quality. Most of the patients in the 14 included studies were in the range of 30-40 years old. As shown in Table 1, almost all COVID-19 patients were in the recovered stage, and most were diagnosed with mild type or moderate type. Eleven studies reported the medical treatments applied for COVID-19 patients [ 22– 31, 34]. In the enrolled studies, commonly used antiviral drugs include ribavirin, interferon, hydroxychloroquine, glucocorticoids, and azithromycin. There are concerns that taking these drugs during COVID-19 infection may have an adverse influence on sperm parameters. In Holtmann et al.’s article, it was suggested that the treatment with lopinavir/ritonavir and hydroxychloroquine would not have a significant impact on sperm parameters since they were only used for a few days [ 25]. And there is no evidence that hydroxychloroquine or azithromycin negatively affects male fertility and sperm parameters [ 41, 42]. The study by Gul et al. also showed that use of favipiravir and hydroxychloroquine had no long-term impairment on male fertility [ 30]. Steroids are recommended to be used in small doses for short periods, and it is believed that short-term use of the drug in small amounts have minimal effect on the male reproductive health [ 43].

ACE2 is a receptor with a high affinity for SARS-CoV-2 that facilitates virus enter human cells with the assistance of TMPRSS2 [ 9, 11]. A number of literatures have shown high expression of ACE2 protein in testicular tissue [ 12, 14], suggesting that the testis may be a target organ for SARS-CoV-2 infection. However, whether the virus can enter testis and damage the testicular tissue, thus affecting spermatogenesis is controversial and inconclusive. Although most literature did not confirm the existence of SARS-CoV-2 virus in semen of COVID-19 male patients [ 22, 26, 44– 47], some studies have detected the virus RNA in semen of men infected with COVID-19. In one study, SARS-CoV-2 was found in the semen of 4 out of 30 COVID-19 patients [ 48]. The four patients were in the acute phase and had severe pneumonia. Moreover, they had a much higher viral load than other patients based on their clinical symptoms and laboratory findings. It is reported that SARS-CoV-2 RNA was detected in semen samples from one out of fifteen COVID-19 patients [ 49]. Patients in this study were asymptomatic or had mild symptoms, and their semen samples were taken no more than 2 weeks from the onset of symptoms [ 49]. The study by Li et al. found that of 38 COVID-19 patients who contributed semen samples, six patients were tested positive for SARS-CoV-2 RNA in their semen, of which four were in acute infection and two were in convalescence [ 50]. These results suggest that SARS-CoV-2 virus may be able to enter the testis and be present in the semen, especially in severe cases with a high viral load. It may take some time for the virus to clear in semen.

Previous studies have reported the testicular pathological findings in patients with COVID-19. Duarte-Neto et al. performed percutaneous autopsy on eleven patients who died of COVID-19 to obtain postmortem testicular samples [ 51]. SARS-CoV-2 viral antigen was detected in Sertoli cells, Leydig cells, spermatids, and fibroblast cells in rete testis in all eleven cases by electron microscopy (EM), immunohistochemistry (IHC) also identified viral particles in multiple cells of testis in four cases, and SARS-CoV-2 RNA was detected in testis tissue in three cases by RT-PCR. Eight of the eleven patients had mild interstitial orchitis, and all cases had hyperemia, interstitial edema, basement membrane thickening, Leydig and Sertoli cell reduction, and reduced spermatogenesis [ 51]. Achua et al. found that SARS-CoV-2 viral particles were detected in testis tissues in 2 of 5 COVID-19 cases [ 52]. Moreover, hematoxylin-eosin (H&E) histomorphology showed impaired spermatogenesis in 3 of 6 COVID-19 patient biopsy cases. H&E stain demonstrated infiltration of interstitial macrophages and leukocytes. Yang et al. conducted postmortem examination on the testes of twelve COVID-19 male cases [ 53]. They identified mild to severe tubular damage in 11 patients, with Sertoli cells swelling, vacuolation and shedding. However, no viral particles were detected by transmission electron microscopy (TEM), and no viral RNA was detected in most cases by RT-PCR [ 53]. In one study by Li et al. [ 54], the autopsy of testicular and epididymal samples from COVID-19 patients showed interstitial edema, congestion, and erythrocyte exudation in the testes and epididymides [ 54]. Moreover, thinning of seminiferous tubules, increased apoptotic cells in tubules, higher concentrations of CD3 ^+^ and CD68 ^+^ in the Leydig cells, and the presence of IgG in tubules were observed [ 54].

These studies suggest that the testis may be susceptible and vulnerable to COVID-19. The SARS-CoV-2 virus may invade multiple testicular cells, including Sertoli cells, spermatogonia, and interstitial cells based on ACE2 and TMPRSS2 expression in these cells. It will cause local immune and inflammatory responses, thus making damage to the structure and function of seminiferous tubules and affecting the local microenvironment of testis and epididymis [ 54]. Especially in severe cases with high viral load, SARS-CoV-2 virus may spread to the reproductive tract through the blood-testis barrier due to the systemic inflammatory response [ 50]. Consequently, spermatogenesis and hormone secretion in COVID-19 patients would be damaged by the virus, leading to male fertility impairment. However, we cannot conclude that SARS-CoV-2 virus can directly invade and attack testicular cells, and it needs more experiments and data to prove.

There are also some other possible mechanisms that contribute to impaired fertility in COVID-19 male patients. Inflammation itself can also cause testicular damage, and orchitis is primarily based on systemic inflammation and secondary autoimmune responses induced by SARS-CoV-2 virus [ 7]. Moreover, epididymitis can coexist with orchitis and lead to side effects such as spermatozoa damage and irregular sex hormone secretion [ 45]. During viral infection, inflammation may cause dysregulation of the hypothalamic-pituitary-gonadal (HPG) axis and subsequently affects sex hormone production and spermatogenesis [ 55, 56]. Complex inflammatory infiltration can cause extensive destruction of germ cells and spermatids [ 57]. Due to hyperactivation of the human immune system after COVID-19, the virus infection can lead to cytokine storms under strong immune stimulus, thus making damage to multiple organs in the body, including the testis [ 58]. In addition, fever, hypoxia, and mental stress during COVID-19 are also important factors impairing male fertility and sperm quality [ 21].

Multiple human systems are affected after COVID-19 infection, but the damage to male reproductive system is not given enough attention. The number of articles exploring the reproductive health of male patients after COVID-19 infection is very limited, and no regular examination or preventive measures have been introduced to protect the reproductive health of male patients. This article is the most up-to-date meta-analysis investigating the association between COVID-19 infection and sperm quality with a large number of COVID patients and a subgroup analysis based on the recovery time. We systemically reviewed and analyzed semen data from 1174 COVID patients, and we conclude that SARS-CoV-2 infection affects male sperm quality, providing evidence for regular examining testicular function in male patients with COVID-19. To date, we have not found any specific methods of fertility protection for COVID-19 patients in the literature. However, according to European Association of Urology Guidelines on Sexual and Reproductive Health [ 59] and our findings, the reproductive health of COVID-19 male patients should be aware of by both physicians and patients, and empirical health promotion could be adopted, such as physical exercise, lifestyle improvement, smoking, and alcohol cessation. Regular semen analysis is also recommended, and when sperm quality is detected to show a significant decline, drug treatment and sperm cryopreservation may be considered to protect and preserve fertility.

The current study has some limitations. First, we did not conduct subgroup analyses on the age or disease severity of patients since the relevant data is limited or inapplicable. Therefore, more studies are needed to refine the study. Second, semen parameters do not fully reflect male fertility. Data on seminal plasma biochemical, sperm DNA integrity, and inflammatory markers are also useful indicators to evaluate semen quality and male fertility. Due to extremely limited data, we are unable to analyze these parameters in this study. In addition, routine semen analysis is somewhat subjective, and the results of each test could be fluctuated. However, semen parameters remain the most common and fundamental evaluation indicators of male reproductive health.

In summary, we found that SARS-CoV-2 infection has a detrimental influence on human sperm parameters, especially on total motility and sperm concentration. Our study indicates that semen quality was significantly decreased within 3 months after COVID-19 infection and maybe recovered after 3 months due to neosperm genesis. The virus may spread to male reproductive system and impair spermatogenesis. This indicates that more consideration should be given to reproductive health in male patients after COVID-19 infection. We suggest these patients receive long-time follow-up and routine screen. More original studies are required to elucidate the effects of SARS-CoV-2 infection on the male fertility.

## 4. Materials and Methods

### 4.1. Search Strategy

This systematic review and meta-analysis was performed following the reporting proposal for MOOSE (Meta-analysis Of Observational Studies in Epidemiology) [ 60] and the PRISMA statement [ 61]. Before screening literature, the review was registered in PROSPERO registry (registration number: CRD42021275823). We searched PubMed, EMBASE, Cochrane Library, Clinicaltrials.gov, CBM, CNKI, and Wanfangdata to obtain all relevant papers through June 10, 2022. We use a search strategy that combines text words with subject headings, and our search terms include “COVID-19,” “2019-nCoV,” “coronavirus disease 2019,” “2019 novel coronavirus,” “severe acute respiratory syndrome coronavirus 2,” “SARS-CoV-2,” “male fertility,” “semen,” “sperm,” “semen quality,” “semen parameters,” “testis,” “reproductive health,” “spermatozoa,” and “germ cell” with language restriction to English and Chinese (Supplementary Table S1).

### 4.2. Study Selection

Studies were included if they [1] were cohort studies or cross-sectional studies; [2] were original human research; [3] involved participants who were diagnosed with COVID-19 by nasopharyngeal swab RT-PCR test; and [4] reported sperm parameters data of COVID-19 male patients and COVID-19 (-) male controls. Studies were excluded if they [1] were letters, reviews, meta analyses, case reports, animal experiments, or basic studies; [2] contained duplicate data or overlapping participants; and [3] had serious quality defects or their full texts were not available.

Two authors (X.C. and J.L.D.) carried out the literature screening process independently. One author (J.L.D.) extracted all data that met the inclusion criteria into an excel spreadsheet, and then another author (X.C.) checked. A third author (M.L.) was consulted in case of any disagreement. Discrepancy on the relevance and quality of literature was resolved by consensus among the three authors after discussion. The following information was extracted: first author, year of publication, country, study design, sample size, mean age, sampling time, and sperm parameters (including semen volume, total sperm number, sperm concentration, total sperm motility, progressive sperm motility, and normal sperm morphology). For papers where original data cannot be found, we sent emails to the corresponding authors to seek the original data.

### 4.3. Data Extraction and Transformation

As shown in Table 2, we extracted the outcomes of six sperm parameters in the nine included articles. We found some literature mainly described the sperm parameters using median and interquartile range (IQR) or range [ 21, 27, 29, 31– 33]. To conduct data synthesis, we converted median to mean and IQR or range to standard deviation (SD) according to Cochrane Handbook [ 62]. Two studies involved COVID-19 patients with different degrees of severity, and their semen data were presented in two or more categories [ 25, 28]. In this condition, we summarized the classes into one COVID-19 case group using formulas proposed by Xiang et al. [ 63]. One study provided semen data in histograms in a figure [ 26]; we extracted the sperm parameters data from the histograms using WebPlotDigitizer (Website: https://automeris.io/WebPlotDigitizer Version: 4.5 Pacifica, California, USA) [ 64].

### 4.4. Data Analysis

We conducted a meta-analysis using Stata 16. Pooled MD and its 95% confidence interval were calculated to evaluate differences in sperm parameters between the COVID-19 male cohort and the uninfected control cohort. Heterogeneity was measured using the Q test, H -statistic and I -squared statistic. I^2^ values ≥ 50% indicated a high level of heterogeneity, and a REM was utilized to analyze the pool results in this condition. Otherwise, a FEM was used. We performed sensitivity analyses to evaluate the stability of our results and to analyze the sources of bias. The funnel plot and Egger’s test were used to assess the potential publication bias. Calculations were performed using StatsToDo: Combine Means and SDs Into One Group Program (combine means SDs (statstodo.com)) and the formula from Xiang et al. [ 63]. We conducted a subgroup analysis based on the recovery time of the COVID-19 patients. It is noted that the recovery time is roughly estimated based on the description of time of semen sampling or patient enrollment in the original articles, mainly on the median time.

Quality assessment of the enrolled studies was conducted by two authors (X.C. and M.L.) using the Newcastle-Ottawa Scale (NOS) [ 65]. The detailed evaluation method is presented in Supplementary Table S2. The NOS score ranges from zero to nine stars, and high-quality studies are rated 6-9 stars.

## Supplementary Materials

Supplementary Materials Supplementary Figure S1: funnel plots for included studies. Supplementary Figure S2: sensitivity analysis for included studies. Supplementary Table S1: searching strategy. Supplementary Table S2: quality assessment of studies included.
