# Supplementary material for: A Systemic Review and Meta-analysis of the Effect of SARS-CoV-2 Infection on Sperm Parameters
Source: Research (Wash D C). 2022 Jul 13;2022:9835731. doi: 10.34133/2022/9835731 (PMC11412417; doi:10.34133/2022/9835731)
Supplement: Supplementary Materials — Supplementary Figure S1: funnel plots for included studies. Supplementary Figure S2: sensitivity analysis for included studies. Supplementary Table S1: searching strategy. Supplementary Table S2: quality assessment of studies included. [file 9835731.f1.docx]

A systemic review and meta-analysis of the effect of SARS-CoV-2 infection on sperm parameters

*Xi Chen, Jinli Ding, Miao Liu, Kai Xing, Peng Ye, Junxia Min, Yan Zhang, Tailang Yin*

**The file includes:**

**Supplementary Figure S1. Funnel Plots for Included Studies**

**Supplementary Figure S2. Sensitivity Analysis for Included Studies**

**Supplementary Table S1. Searching strategy**

**Supplementary Table S2.** **Quality Assessment of Studies Included**

**
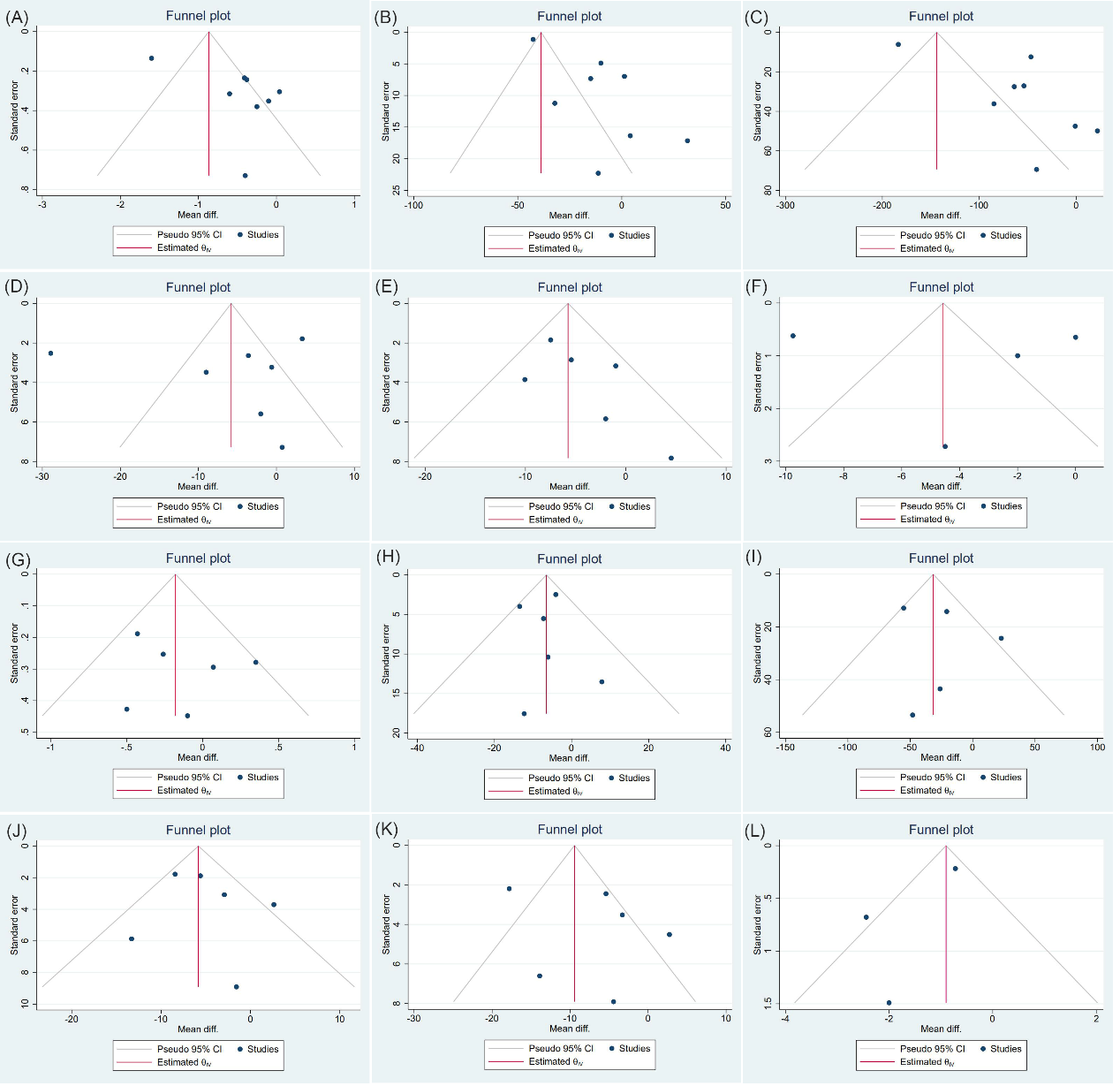
**

**Supplementary Figure S1. Funnel Plots for Included Studies.** (A-F). Funnel plots in case-control studies: (A). Funnel plot of semen volume; (B). Funnel plot of sperm concentration; (C). Funnel plot of total sperm count; (D). Funnel plot of progressive sperm motility; (E). Funnel plot of total sperm motility; (F). Funnel plot of normal sperm morphology. (G-L). Funnel plots in pre to post COVID-19 studies: (G). Funnel plot of semen volume; (H). Funnel plot of sperm concentration; (I). Funnel plot of total sperm count; (J). Funnel plot of progressive sperm motility; (K). Funnel plot of total sperm motility; (L). Funnel plot of normal sperm morphology.


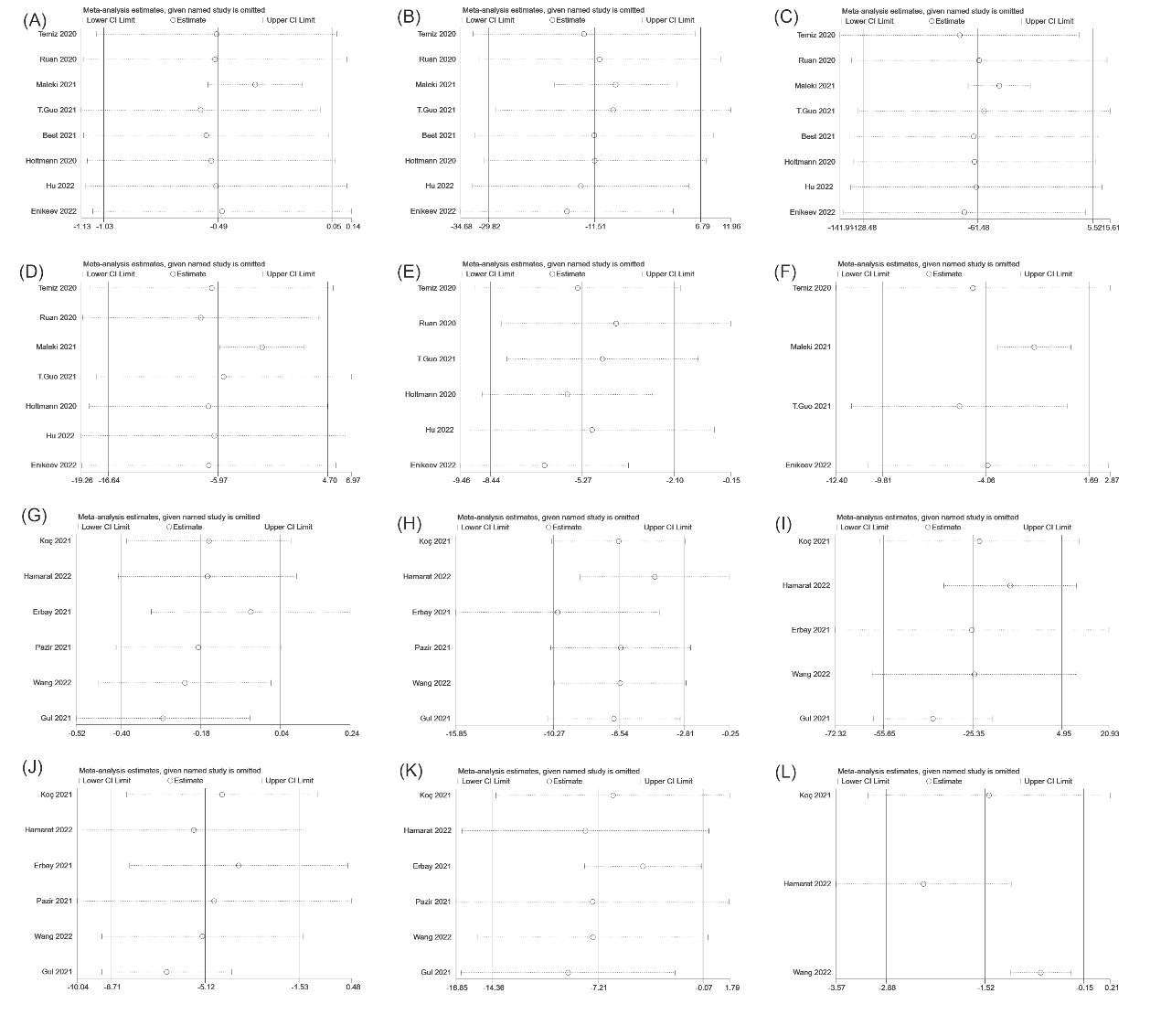


**Supplementary Figure S2 Sensitivity Analysis for Included studies.** (A-F). Sensitivity analysis in case-control studies: (A). Sensitivity analysis of semen volume; (B). Sensitivity analysis of sperm concentration; (C). Sensitivity analysis of total sperm count; (D). Sensitivity analysis of progressive sperm motility; (E). Sensitivity analysis of total sperm motility; (F). Sensitivity analysis t of normal sperm morphology. (G-L). Sensitivity analysis in pre to post COVID-19 studies: (G). Sensitivity analysis of semen volume; (H). Sensitivity analysis of sperm concentration; (I). Sensitivity analysis of total sperm count; (J). Sensitivity analysis of progressive sperm motility; (K). Sensitivity analysis of total sperm motility; (L). Sensitivity analysis of normal sperm morphology.

**Supplementary Table S1: Search Strategy**

"COVID-19"[MeSH Terms]

COVID-19

COVID19

2019-nCoV

coronavirus disease 2019

2019 Novel Coronavirus

"SARS-CoV-2"[MeSH Terms]

SARS-CoV-2

severe acute respiratory syndrome coronavirus 2

"Fertility"[MeSH Terms]

Fertility

Male fertil*

"Fertility Preservation"[MeSH Terms]

Fertility Preservation

"Reproductive health"[MeSH Terms]

reproductive health

male reproducti*

"semen"[MeSH Terms]

Semen*

Semen quality

Semen parameters

"Semen analysis"[MeSH Terms]

Semen analysis

"Spermatozoa"[MeSH Terms]

Sperm*

Sperm quality

Sperm parameters

"Testis"[MeSH Terms]

Testis

Testicular function

"Germ Cells"[MeSH Terms]

| Pubmed | ("COVID-19"[MeSH Terms] OR "COVID-19"[Title/Abstract] OR "COVID、19"[Title/Abstract] OR "2019-nCoV"[Title/Abstract] OR "coronavirus disease 2019"[Title/Abstract] OR "2019 novel coronavirus"[Title/Abstract] OR "SARS-CoV-2"[MeSH Terms] OR "SARS-CoV-2"[Title/Abstract] OR "severe acute respiratory syndrome coronavirus 2"[Title/Abstract]) AND ("Fertility"[MeSH Terms] OR "Fertility"[Title/Abstract] OR "male fertil*"[Title/Abstract] OR "Fertility Preservation"[MeSH Terms] OR "Fertility Preservation"[Title/Abstract] OR "Reproductive health"[MeSH Terms] OR "Reproductive health"[Title/Abstract] OR "male reproducti*"[Title/Abstract] OR "Semen"[MeSH Terms] OR "semen*"[Title/Abstract] OR "semen quality"[Title/Abstract] OR "semen parameters"[Title/Abstract] OR "Semen analysis"[MeSH Terms] OR "Semen analysis"[Title/Abstract] OR "Spermatozoa"[MeSH Terms] OR "sperm*"[Title/Abstract] OR "sperm quality"[Title/Abstract] OR "sperm parameters"[Title/Abstract] OR "Testis"[MeSH Terms] OR "Testis"[Title/Abstract] OR "testicular function"[Title/Abstract] OR "Germ Cells"[MeSH Terms]) | 982 |
| --- | --- | --- |
| Embase | #1 'coronavirus disease 2019':ti,ab,kw OR '2019 ncov':ti,ab,kw OR 'covid 19':ti,ab,kw OR 'sars cov 2':ti,ab,kw OR 'severe acute respiratory syndrome coronavirus 2':ti,ab,kw OR '2019 novel coronavirus':ti,ab,kw  #2 fertility:ti,ab,kw OR 'fertility preservation':ti,ab,kw OR semen:ti,ab,kw OR sperm:ti,ab,kw OR spermatozoon:ti,ab,kw OR 'germ cell':ti,ab,kw OR 'sperm quality':ti,ab,kw OR 'semen parameters':ti,ab,kw OR 'testis':ti,ab,kw OR 'male genital system':ti,ab,kw  #1 AND #2 | 868 |
| Clinicaltrials | Search of: Fertility OR "Reproductive health" OR "male reproduction" OR semen OR sperm OR "semen analysis" OR "semen quality" OR "sperm parameters" OR "testis" OR "germ cells" \| COVID-19- List Results - ClinicalTrials.gov | 30 |
| Cochrane library | Using "Fertility OR 'Reproductive health' OR 'male reproduction' OR semen OR sperm OR 'semen analysis' OR 'semen quality' OR 'sperm parameters' OR 'testis' OR 'germ cells' + Report Results" in the Cochrane Library's special website https://covid-19.cochrane.org/ to screen target literature. | 540 |
| CBM | ("sperm" OR "semen" OR "male fertility" OR "reproduction" OR "reproductive system" OR "sperm parameters" OR "semen quality" OR "testis" OR "testicular function") AND ("SARS-CoV-2" OR "coronavirus" OR "2019-nCOV" OR "novel coronavirus" OR "COVID-19") | 186 |
| CNKI | ((SARS-CoV-2) OR (Novel Coronavirus) OR (2019-nCOV) OR (COVID-19)) AND ((Sperm) OR (Sperm) OR OR (Sperm parameter) OR (Semen quality) OR (testis) OR (Reproduction) OR (Fertility)) | 87 |
| Wanfang data | (Theme: SARS-CoV-2 OR 2019-ncov OR COVID-19 OR (Novel Coronavirus) OR 2019-nCOV) and theme: (sperm OR semen OR testis OR reproduction OR fertility)) and Date:2019-* | 272 |

**Supplementary Table S2: Quality Assessment of Studies Included**

| Study | The selection of the study | | | | The comparability of the study | The outcome in the study | | | Total scores |
| --- | --- | --- | --- | --- | --- | --- | --- | --- | --- |
|  | Was the exposed cohort representative | The selection of the non- exposed cohort | Ascertainment of exposure | Demonstration that outcome of interest was not present at start of study | The comparability of groups on the basis of the design or analysis | The assessment of the outcome | Was follow-up long enough for the outcome to occur? | The adequacy of follow-up |  |
| Temiz 2020 | 1 | 1 | 1 | 1 | 2 | 1 | 1 | 1 | 9 |
| Ruan 2020 | 1 | 1 | 1 | 1 | 2 | 1 | 1 | 1 | 9 |
| Maleki 2021 | 1 | 1 | 1 | 1 | 1 | 1 | 1 | 1 | 8 |
| T.Guo 2021 | 1 | 1 | 1 | 1 | 2 | 1 | 1 | 1 | 9 |
| Best 2021 | 1 | 1 | 1 | 1 | 2 | 1 | 1 | 1 | 9 |
| Holtmann 2020 | 1 | 1 | 1 | 1 | 0 | 1 | 1 | 1 | 7 |
| Hu 2022 | 1 | 1 | 1 | 1 | 1 | 1 | 1 | 1 | 8 |
| Enikeev 2022 | 1 | 1 | 1 | 1 | 0 | 1 | 1 | 1 | 7 |
| Pazir 2021 | 1 | 1 | 1 | 1 | 2 | 1 | 1 | 1 | 9 |
| Koç 2021 | 0 | 1 | 1 | 0 | 2 | 1 | 1 | 1 | 7 |
| Erbay 2021 | 0 | 1 | 1 | 0 | 2 | 1 | 1 | 1 | 7 |
| Hamarat 2022 | 0 | 1 | 1 | 0 | 2 | 1 | 1 | 1 | 7 |
| Wang 2022 | 0 | 1 | 1 | 0 | 2 | 1 | 1 | 1 | 7 |
| Gul 2021 | 1 | 1 | 1 | 1 | 2 | 1 | 1 | 1 | 9 |

For Selection:

Ten publications received 4 stars (each reported patients in the exposed cohort tested positive for SARS-CoV-2 by RT-PCR; each reported that the non-exposed cohorts were from the same population as the exposed cohort; each reported that the non-exposed group had no history of infertility).

Four publications only received 2 stars for including patients having applied to andrology clinic for infertility or reproductive technology treatment, and no declaration of excluding patients with some testicular diseases, such as varicocele and undescended testis. They cannot be truly representative of the semen quality of COVID-19 recovered male patients for their initial semen quality may have declined.

For Comparability:

Ten publications received 2 stars (each controlled important factors such as age, BMI, the proportion of smokers, and no significant difference was found).

Maleki 2021 received 1 star for having evaluating the relevant difference, but significant difference was found in BMI between the exposed and non-exposed groups.

Hu 2022 received 1 star for not evaluating the relevant difference, and BMI, smoking status of the control group were not provided.

Enikeev 2022 received 0 star for significant difference was found in age between the exposed and non-exposed groups.

Holtmann 2020 did not earn a star for not evaluating the relevant difference, and age between two cohorts was obviously different.

For Exposure:

All publications received 3 stars (each reported semen analysis performed in accordance to the WHO guidelines; the follow-up time was enough to observe the semen quality of COVID-19 recovered patients; complete follow-up).
